# Systemic Lupus Erythematosus Presenting as Longitudinally Extensive Transverse Myelitis and Nephritis: A Case Report

**DOI:** 10.7759/cureus.2402

**Published:** 2018-04-01

**Authors:** Abdul S Zahid, Ayesha Mubashir, Samir A Mirza, Iftikhar H Naqvi, Abu Talib

**Affiliations:** 1 Civil Hospital, Karachi, Dow University of Health Sciences (DUHS), Karachi, Pakistan; 2 Department of Internal Medicine, Dow University of Health Sciences (DUHS), Karachi, Pakistan; 3 Civil Hospital, Dow University of Health Sciences (DUHS), Karachi, Pakistan

**Keywords:** sle, lupus nephritis, acute extensive longitudinal myelitis, sle

## Abstract

Systemic lupus erythematosus (SLE) is an autoimmune disorder that can potentially affect any organ. It usually presents between the ages of 15 and 45 with 9:1 ratio of female to male patients. Its clinical manifestations vary among people of different ethnicities. Longitudinally extensive transverse myelitis (LETM) is a rare life-threatening complication of SLE. We, herein, report a case of 26-year-old male diagnosed with LETM along with lupus nephritis.

The patient presented with high-grade fever associated with chills and burning micturition followed by progressive bilateral lower limb weakness and urinary retention. His physical examination showed decreased bilateral lower limb power, absent reflexes, and mute plantars. His abdominal reflexes were also found to be absent and sensory level was identified at T10. T2 weighted magnetic resonance imaging (MRI) of the dorsal spine showed hyper-intense signals between T5-L1 suggestive of extensive longitudinal myelitis. Renal biopsy confirmed the presence of lupus nephritis stage III + V. Anti-nuclear antibodies (ANA) were reactive and anti-dsDNA was positive, indicative of SLE as the underlying cause of his clinical manifestations.

The treatment strategy proved to be beneficial in our patient. However, there is still a vast gap between understanding the mechanisms of self-reactive diseases such as SLE and the appropriate therapeutic approach. As Pakistan’s first documented case of Lupus Myelitis, we hope to delve deeper into this matter.

## Introduction

Systemic lupus erythematosus (SLE) is an autoimmune disease that affects approximately 5 million people worldwide [1]. Despite its scattered global distribution it is more commonly seen in Asians and African Americans [1]. Patients usually present with pain, fatigue, fever, rash and arthralgia or arthritis as the initial symptoms [1]. Clinical manifestations of lupus vary and the symptoms can range in severity and location of the organs involved.

One important presentation of SLE is lupus nephritis (LN). LN commonly presents with increased serum creatinine, microscopic hematuria, urinary cast and proteinuria. LN is found in 20–50% of all patients and its prevalence is higher in SLE patients of Asian ethnicity [2]. A total of 50–60% patients present with the sequelae during the course of their disease, compared to 30–38% of Caucasian patients who show these manifestations [2]. According to a study presented by Ishaq et al., the incidence is lower in Pakistan compared to other countries, with about 22.8% patients developing LN [1]. The extent and pattern of renal involvement is divided into various classes by International Renal Society and World Health Organisation (WHO) who have given recommendations for the treatment. The treatment response may vary from patient to patient.

Lupus myelitis (LM) is another rare presentation of SLE, occurring in 1–2% of patients, hence there have been only a few cases documented worldwide. This usually presents as acute transverse myelitis, which can present as ‘complete’—where it can involve limbs bilaterally and both sensory and motor deficits are seen below the spinal level—or ‘partial’—where there is unilateral and patchy involvement with mild weakness [3]. Another relatively uncommon form is called longitudinally extensive transverse myelitis (LETM), which is defined as involvement of three or more continuous vertebral segments of the spinal cord.

Here we present an intriguing case of a young man with SLE who developed LETM along with LN. To the best of our knowledge, this is also the first documented case of LETM in Pakistan. Given the scarcity of this particular manifestation, the response to the following treatment is of paramount importance to the management of such patients in the future.

## Case presentation

A 26-year-old male presented to the neurology outpatient department (OPD) with an eight-year history of SLE and one-year history of hypertension. He was taking oral prednisone 5 mg and indomethacin 25 mg since 2009 and took these medications for two years before discontinuing them. He complained of undocumented high-grade fever associated with chills and burning micturition for four days for which he took antipyretics and ciprofloxacin 500 mg. This was followed by progressive bilateral lower limb weakness, urinary retention, constipation and thoracolumbar pain for two days.

On presentation, the patient was afebrile, responsive, with stable vital signs. On examination, his bilateral lower limb power was 0/5, absent reflexes and mute planters. His abdominal reflexes were also found to be absent; his sensory level was identified at T10. Cranial nerves and cerebellum were intact. The patient was also observed to have scarring alopecia.

Laboratory studies revealed mild microcytic anemia (hemoglobin 9 g/dL; normal 14.0–18.0 g/dL), normal white blood cells, platelets (219 x 10^3^/µL; normal 150–400 x 10^3^/µL), erythrocyte sedimentation rate (ESR) 79 mm/hour, serum creatinine 0.6 mg/dL (normal 0.5–1.40), C-reactive protein (CRP) 8.8 mg/L, prothrombin time (PT) 10.3 s, activated partial thromboplastin time (aPTT) 29 s, international normalized ratio (INR) 1.9 (normal 0.8–1.1). Serum albumin was low at 2.9 g/dL (normal 3.5–5.0).

Hepatitis B virus (HBV), hepatitis C virus (HCV) and human immunodeficiency virus (HIV) serology were found to be negative. Antiphospholipids antibody (APLA) were negative. Antinuclear antibodies (ANA) were reactive (58U), anti-dsDNA positive at 197.5 IU/mL, anticardiolipin IgG equivocal at 10.90 GLP/mL. Hypocomplimentemia (C3 0.6 g/L; normal 0.8–1.6, C4 0.05 g/L; normal 0.12–0.36) was also identified. Antiaquaporin IgG was negative.

A lumbar puncture revealed increased protein in the CSF 130 mg% (normal 20–40%), while glucose and chloride were within normal ranges.

**Figure 1:**
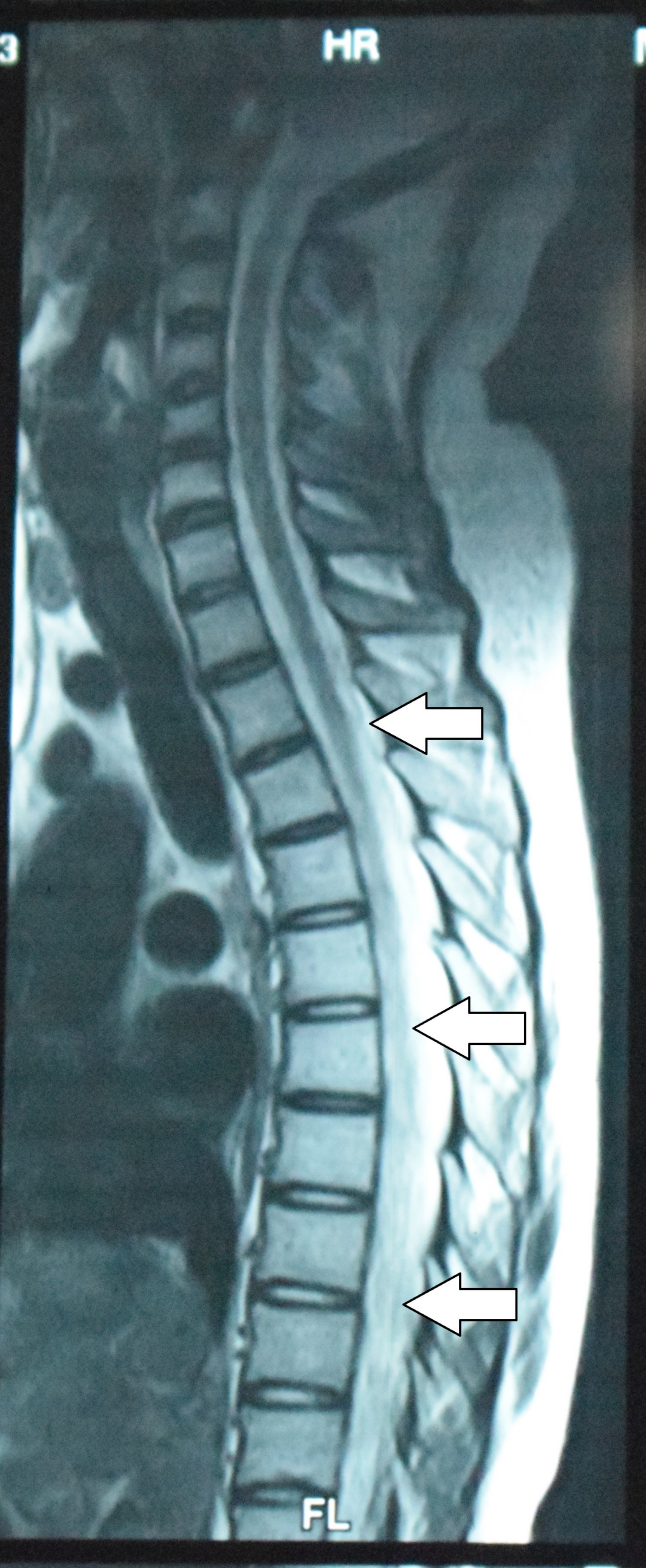
Spinal MRI (saggital) of the dorsal column. T-2 weighted image shows hyper-intense signals between T5-L1 (arrows). MRI: Magnetic resonance imaging.

Magnetic resonance imaging (MRI) dorsal spine showed hyper-intense signals between T5-L1 suggestive of longitudinally extensive transverse myelitis best appreciated on T-2 weighted image (Figure 1). Conus medullaris and filum terminale were found to be unremarkable.

Ultrasound abdomen showed a calculus in the left kidney (0.6 cm). Chest X-ray, pelvic ultrasound and echocardiogram were normal.

Blood culture was positive for klebsiella growth and urine culture was positive for E. coli. Based on sensitivity reports, the patient was started on amikacin 500 mg and ceftriaxone 2 g for one week.

Treatment with steroid pulse therapy (1 g of methylprednisolone intravenously per day for five continuous days) was given but showed no neurological improvement. Oral prednisolone 1 mg/kg was then started and its dose was gradually tapered. Five sessions of plasmapheresis were also done. The patient was started on a monthly dose of intravenous (IV) cyclophosphamide of 1 g (monthly for six months). Upon discharge after a month, the patient showed improvement and his bilateral lower limb power increased to 2/5.

One month later, he presented to the medical OPD with complaints of fever of 108°F, burning micturition for two weeks, shortness of breath, malar erythematous rash sparing the nasolabial folds with itching, photosensitivity, oral ulcers and dysphagia for one week.

On examination, his blood pressure (BP) was 130/100 mmHg, sensation intact, bilateral lower limb reflexes normal, power 2/5 and positive bilateral Babinski sign.

Clinical findings included mild microcytic anemia (hemoglobin 9 g/dL; normal 13–18), total leukocyte count 19.4 x 10^3^/µL (normal 4–11 x 10^3^/µL), platelets 284 x 10^3^/µL (normal 150–400 x 10^3^/µL), serum creatinine 0.8 mg/dL (normal 0.6–1.12 mg/dL), PT 11.4s, aPTT 27.5s, INR 1.43, total protein 6.1 g/dL (normal 6.4–8.3), A/G ratio 0.69 (normal 0.8–2.0), total serum calcium 7.3 mg/dL (normal 8.5–10.2), serum magnesium 0.1 mEq/L (normal 1.5–2.5), total iron binding capacity 121 µg/dL (normal 240–450). Serum low-density lipoproteins (LDL) (100 mg/dL; normal <100 mg/dL).

Urinalysis showed red cells 16–20/HPF, pus cells 8–10/HPF, occasional epithelial cells, protein 3+, leukocytes +/UL, blood 2+, amorphous urate 3+. Urine protein/24 hours was found to be 3280 mg and 24-hour urine output was recorded to be 4000 mL. No bacterial growth was observed upon urine culture. These findings were consistent with lupus nephritis. Renal biopsy confirmed the presence of lupus nephritis stage III + V. Immunofluorescence showed diffuse mesangial, membranous positivity of C1q (+++), IgG (+), C3 (+) and weakly positive for IgA and IgM.

**Figure 2:**
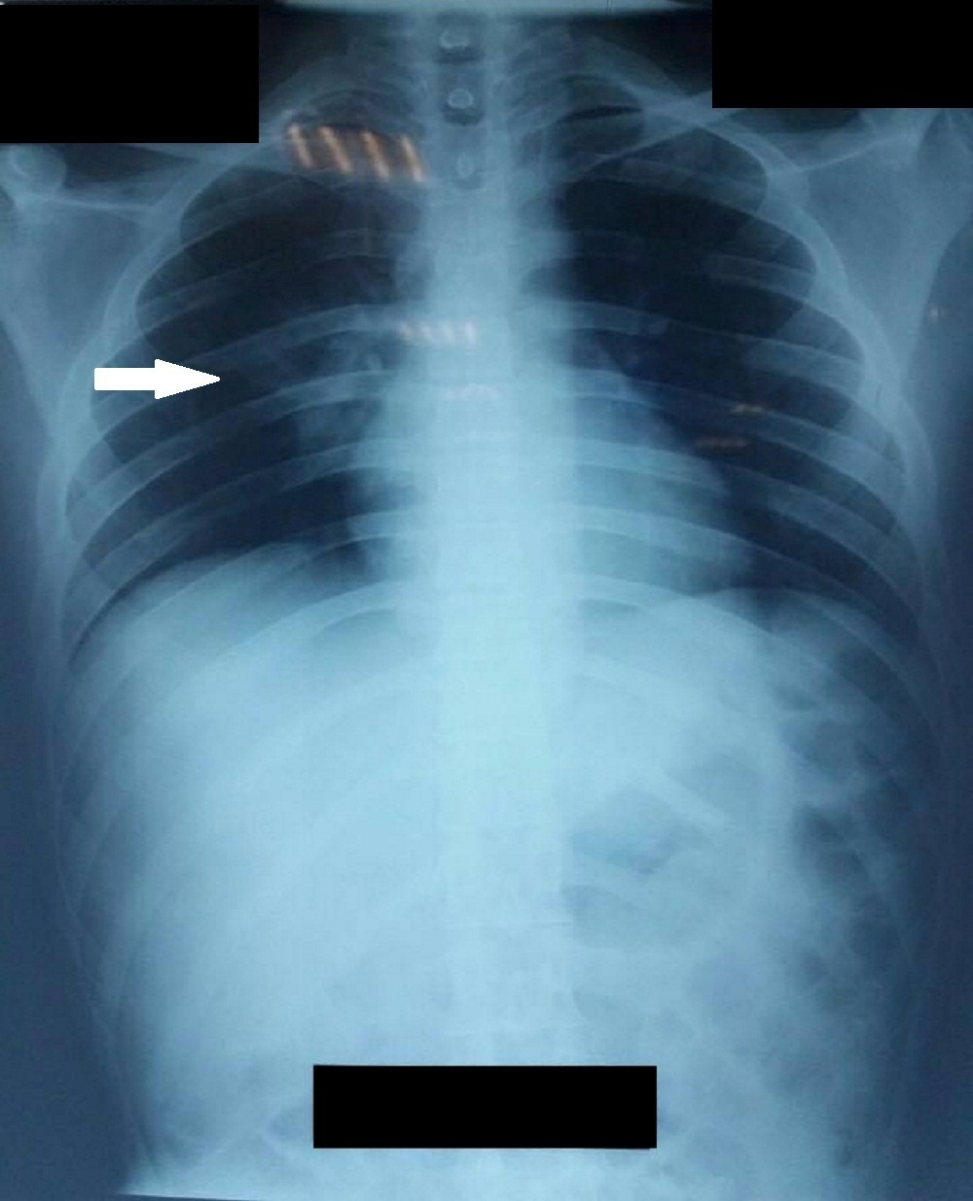
Chest radiograph showing opacities medially in the right lobe.

Chest X-ray showed opacity medially in the right lung as shown in Figure 2. The patient was diagnosed with pneumonia. Blood cultures were negative for bacterial growth and hence the patient was started on piperacillin/tazobactam 4.5 gm thrice a day for 10 days. He was then started on methylprednisolone 1 g IV for five continuous days. Oral prednisolone 5 mg was started after completion of methylprednisolone therapy and its dose was gradually tapered. The second dose of 1 g IV cyclophosphamide was then administered in accordance with the American College of Rheumatology (ACR) guidelines after which he was started on a maintenance therapy with azathioprine. He was also started on hydroxychloroquine 400 mg, lisinopril 10 mg, and atorvastatin 10 mg.

The patient was called in for a follow-up after a month. On follow-up examination he was afebrile, his sensation was intact, bilateral lower limb reflexes normal, power 4/5 and positive Babinski sign. The patient was able to stand with support. His dermal condition had also improved. He, however, still complained of occasional urinary incontinence.

Lab tests were repeated and showed CRP 2.7 mg/dL, serum creatinine 0.5 mg/dL and blood urea nitrogen (BUN) 12 mg/dL.

Urinalysis showed the presence of red cells 1–2/hpf, pus cells 21–25/hpf, amorphous urates +, protein 1+, bile salts ++ and leucocytes ++/UL.

## Discussion

SLE is a multi-system disorder that can occur in any population of the world. The spectrum of clinical manifestations and their severity, however, vary between different ethnicities. While most of these are true representations of biological, environmental and socio-economical differences, some of them are merely the result of lack of good research data on SLE in the developing world.

There is no consistent pattern of organ damage in the developing world but the musculoskeletal, central nervous system and renal system seem to be the most affected. LN is one of the most salient and prevalent manifestations of SLE found in Asians, Latin Americans and Africans. According to a study done by Rabbani et al. [4], grade 3 nephritis was found in 64% and grade 5 found in 17% of the patients who underwent renal biopsy for LN and admitted to a tertiary care hospital in Karachi. LN is associated with active SLE and poor prognosis. According to a recent study on Hispanic patients by Riveros et al. [5], male patients with SLE had a higher prevalence of LN and LA positivity compared to females. Males patients were also found to be more resistant to treatment, had more co-morbidities and presented higher mortality.

LETM, on the other hand, is a severe and rare neuropsychiatric manifestation of SLE and occurs in only 1–3% of SLE patients. The first case series of SLE related LM was reported by Tellez-Zenteno et al. in 2001 [6]. Astonishingly, no case of LETM has been reported from Pakistan. This could be due to lack of awareness on behalf of the physicians or an inadequate research on the subject.

A study done by Bhat et al. [7] reviews possible etiologies of transverse myelitis comprehensively of pertaining articles from 1981 to 2009. According to Bhat et al., 20–24% of all infectious causes of TM are attributed to viral etiology. These include Coxsackie virus, Hepatitis A virus, polio, Herpes simplex virus amongst many others. Our patient's blood culture was positive for Klebsiella and E. coli, which are less likely causes of TM and thus were ruled out.

Furthermore, LETM is typically associated with Neuromyelitis Optica Syndrome (NMOS). However, our patient tested negative for antiaquaporin antibody which is the most important serological marker for NMO. He also did not meet the criteria put forth by the 2015 guidelines of International Panel of Neuromyelitis Optica Diagnosis (IPND), and thus NMO was also ruled out.

Antiphospholipid syndrome is also thought to accompany SLE and can cause difficulty in isolating the cause of myelitis in many patients. Our patient tested negative for aPLA thus favouring SLE to be the cause of LETM. Furthermore, it is also observed that most of the SLE patients who present with TM have a detectable sensory deficit at the thoracic level which is consistent with the findings in our patient [7].

A case of LETM with favourable outcomes has been reported by Mula et al. Patient’s anticardiolipin antibodies and APL tested negative which is consistent with the findings in our patient [8]. Their patient also underwent the same treatment regimen that is synchronized plasmapheresis combined with aggressive immunosuppressant therapy with cyclophosphamide and oral prednisone. After a follow-up of four months, the patient showed complete sensory recovery, improvement in motor function and occasional urinary urgency.

Current treatment guidelines as provided by the ACR include the use of immunosuppressants such as cyclophosphamide (CYC), mycophenolatemofetil (MMF) and azathioprine (AZA). CYC and MMF are considered equivalent based on a meta-analysis [9]. But long-term studies with CYC are abundant as compared to those with MMF. These guidelines have been derived from studies conducted in western populations, whose risk factors and general patterns of disease may be different from those in developing countries; locally derived thresholds in South Asia differ from western populations. Unfortunately, no such study has been done in South Asia, therefore further research is imperative to assess the response of South Asian population to different treatment regimens.

While these drugs have shown a significant reduction in the SLE flare, they have also been reported to have led to bone marrow suppression and serious infections involving urinary tract and chest. Our patient also developed pneumonia. This could have been a manifestation of SLE itself, but there is a high chance that it developed because of the administration of an immunosuppressant. Patients are also likely to be infected by TB, HIV, CMV and nontyphoidal salmonellosis.

Furthermore, risk factors for poor outcome are renal disease and occurrence of a major infection [10], which are the biggest conferrers of mortality in SLE patients. Others include low C3, hematuria, hypertension, and raised creatinine, lack of remission, low patient compliance, socio-economic state and lack of awareness. Our patient showed marked recovery on follow-up, this could be partly because of the absence of APL. Nonetheless, vigorous monitoring and continuous follow-ups will be required to keep the health of the patient in check and prevent relapses.

The future of SLE patients in developing countries like Pakistan can be improved by a joint effort by the government and private sector by developing algorithms for diagnosis based on the available resources, initiation of awareness and drug donation programs.

## Conclusions

In conclusion, we have described the concurrence of two important complications in the same patient with SLE. The paucity of data regarding etiology and treatment of LN and LETM makes them a life-threatening complication.

## References

[1] Ishaq M, Nazir L, Riaz A, Kidwai SS, Haroon W, Siddiqi S (2013). Lupus, still a mystery: a comparison of clinical features of Pakistani population living in suburbs of Karachi with other Asian countries. J Pak Med Assoc.

[2] Yap DY, Chan TM (2015). Lupus nephritis in Asia: clinical features and management. Kidney Dis.

[3] Ford B, Tampieri D, Francis G (1992). Long-term follow-up of acute partial transverse myelopathy. Neurology.

[4] Rabbani MA, Siddiqui BK, Tahir MH, Ahmad B, Shamim A, Shah S MA, Ahmad A (2004). Systemic lupus erythematosus in Pakistan. Lupus.

[5] Riveros Frutos A, Casas I, Rúa-Figueroa I (2017). Systemic lupus erythematosus in Spanish males: a study of the Spanish Rheumatology Society Lupus Registry (RELESSER) cohort. Lupus.

[6] Tellez-Zenteno JF, Remes-Troche JM, Negrete-Pulido RO, Dávila-Maldonado L (2001). Longitudinal myelitis associated with systemic lupus erythematosus: clinical features and magnetic resonance imaging of six cases. Lupus.

[7] Bhat A, Naguwa S, Cheema G, Gershwin ME (2010). The epidemiology of transverse myelitis. Autoimmun Rev.

[8] Mula M, Bolamperti L, Varrasi C (2009). SLE-related longitudinal myelitis with favorable outcome. Can J Neurol Sci.

[9] Touma Z, Gladman DD, Urowitz MB, Beyene J, Uleryk EM, Shah PS (2011). Mycophenolatemofetil for induction treatment of lupus nephritis: a systematic review and metaanalysis. J Rheumatol.

[10] Dhir V, Aggarwal A, Lawrence A, Agarwal V, Misra R (2012). Long-term outcome of lupus nephritis in Asian Indians. Arthritis Care Res (Hoboken).

